# PFASUM: a substitution matrix from Pfam structural alignments

**DOI:** 10.1186/s12859-017-1703-z

**Published:** 2017-06-05

**Authors:** Frank Keul, Martin Hess, Michael Goesele, Kay Hamacher

**Affiliations:** 1 Computational Biology and Simulation, Department of Biology, Technische Universität Darmstadt, Schnittspahnstraße 2, Darmstadt, 64287 Germany; 2 Graphics, Capture and Massively Parallel Computing, Department of Computer Science, Technische Universität Darmstadt, Rundeturmstraße 12, Darmstadt, 64283 Germany

**Keywords:** Substitution matrix, PFASUM, Homologous sequence search, Sequence alignment

## Abstract

**Background:**

Detecting homologous protein sequences and computing multiple sequence alignments (MSA) are fundamental tasks in molecular bioinformatics. These tasks usually require a substitution matrix for modeling evolutionary substitution events derived from a set of aligned sequences. Over the last years, the known sequence space increased drastically and several publications demonstrated that this can lead to significantly better performing matrices. Interestingly, matrices based on dated sequence datasets are still the de facto standard for both tasks even though their data basis may limit their capabilities.

We address these aspects by presenting a new substitution matrix series called PFASUM. These matrices are derived from Pfam seed MSAs using a novel algorithm and thus build upon expert ground truth data covering a large and diverse sequence space.

**Results:**

We show results for two use cases: First, we tested the homology search performance of PFASUM matrices on up-to-date ASTRAL databases with varying sequence similarity. Our study shows that the usage of PFASUM matrices can lead to significantly better homology search results when compared to conventional matrices. PFASUM matrices with comparable relative entropies to the commonly used substitution matrices BLOSUM50, BLOSUM62, PAM250, VTML160 and VTML200 outperformed their corresponding counterparts in 93% of all test cases. A general assessment also comparing matrices with different relative entropies showed that PFASUM matrices delivered the best homology search performance in the test set.

Second, our results demonstrate that the usage of PFASUM matrices for MSA construction improves their quality when compared to conventional matrices. On up-to-date MSA benchmarks, at least 60% of all MSAs were reconstructed in an equal or higher quality when using MUSCLE with PFASUM31, PFASUM43 and PFASUM60 matrices instead of conventional matrices. This rate even increases to at least 76% for MSAs containing similar sequences.

**Conclusions:**

We present the novel PFASUM substitution matrices derived from manually curated MSA ground truth data covering the currently known sequence space. Our results imply that PFASUM matrices improve homology search performance as well as MSA quality in many cases when compared to conventional substitution matrices. Hence, we encourage the usage of PFASUM matrices and especially PFASUM60 for these specific tasks.

**Electronic supplementary material:**

The online version of this article (doi:10.1186/s12859-017-1703-z) contains supplementary material, which is available to authorized users.

## Background

The detection of homologous protein sequences and the construction of protein multiple sequence alignments (MSA) are two of the most fundamental tasks in modern bioinformatics to analyze evolutionary relationships or to infer functional information. Search tools (BLAST [3], FASTA [33], and PSI-BLAST [4]) or MSA algorithms (MUSCLE [14] and MAFFT [28]) require a scoring model to represent evolutionary insertion, deletion and substitution events. While the former two are usually modeled by a specific gap penalty model [5, 11, 44], amino acid substitution events are represented by substitution matrices.

For each two different types of amino acids *A* and *B*, these matrices provide a score which represents the likelihood of amino acid *A* mutating to amino acid *B* in relation to independent evolution. The likelihood of preserving an amino acid type is represented on the diagonal of a substitution matrix. These odds-ratios are normally derived by counting amino acid substitution frequencies in (automatically) aligned sets of sequences. Notably, these sets are usually filtered to remove all parts containing gaps and only focus on highly conserved regions which potentially discards valuable information.

Over the last years, several types of substitution matrices have been developed which differ in construction methodology, underlying data base and field of application. The Point Accepted Mutation matrices (PAM) [10] represent amino acid mutation probabilities for specific evolutionary distances generated through Markov chain models. Starting from an initial distance of 1% amino acid changes on average for the construction of the transition matrix, evolutionary distance is captured in point accepted mutations, ranging from 1 (PAM1) to *n* (PAM*n*). Hereby, the transition matrix is multiplied *n*-times with itself to obtain the *n*-step Markov chain necessary for PAM*n*. The PAM1 probabilities are derived from 1572 amino acid changes between very closely related sequences. Even though this dataset is very small and quite old, PAM matrices are still commonly used for the detection and alignment of closely related sequences. They cannot, however, reliably detect remote homologs [23] or align sequences with low similarity.

Based on the PAM series, further matrices have been developed covering a larger and/or more diverse sequence space [19, 26]. One of the most recent PAM siblings is the VTML matrix series [30, 31] which was originally developed for a better detection of remote homologs but is also used to construct high quality MSAs [14]. VTML matrices are constructed by iteratively estimating evolutionary distances and substitution rates from a set of pairwise sequence alignments using a maximum likelihood estimator. Dayhoff’s model is used as the initial rate matrix. The pairwise alignments are obtained by randomly sampling two pre-aligned sequences from each protein family in the SYSTERS database [29]. This dataset is much larger and more diverse compared to PAM which allows VTML matrices to provide a more reliable detection of remote homologs. However, only pairwise alignments are considered to prevent bias from oversampling and the covered sequence space is still rather small compared to the sequence space available today with only 2.7 million amino acid pairs considered in the VTML generation.

One of the most commonly used matrix series are the BLOSUM matrices [22] which are derived from highly conserved but also distantly related amino acid blocks [21]. To prevent bias from redundant information, similar sequences are clustered. Instead of counting all substitution frequencies fully, they are weighted by the cluster sizes of their corresponding sequences. The matrix numbers, e.g. BLOSUM62, indicate the chosen similarity threshold for the sequence clustering step. The BLOSUM50 and BLOSUM62 matrices are the *de facto* standard for homology search tasks, e.g. in BLAST, PSI-BLAST and FASTA. They are also commonly used for MSA construction and are available as standard parameter in most alignment programs. However, the originating database is quite small with 22.2 million amino acid pairings in its 1992 release and the matrices are known to be substantially biased due to implementation problems [25, 41]. Similar to the PAM series, BLOSUM matrices have also been used as basis for other matrices. Examples are the OPTIMA5 [27] and PBLOSUM [40] matrices which are reported to show improved substitution matrix performance. However, these still rely on the same outdated and small sequence data as BLOSUM.

The selection of the right substitution matrix and corresponding gap parameters is an intricate task essential in homology search, construction of MSAs and phylogeny [1, 18, 24, 35, 37]. While most of the commonly used matrices deliver reasonable results, there are still situations where the sensitivity of these matrices is not sufficient. A possible explanation for this issue is that these matrices are derived from too small and similar datasets that do not contain enough variety. This is in concordance with common knowledge that matrices derived from a larger and more diverse sequence space can lead to significantly better homology search performance [25, 35].

In this paper, we address this sensitivity issue by presenting a novel type of substitution matrix based on structural alignments. Our **Pf**am **su**bstitution **m**atrix (PFASUM) series is derived from the manually curated Pfam seed alignments (version 29.0) [16] using a novel algorithm. Thus, our PFASUM matrices rely on state-of-the-art expert ground truth data which covers a much larger and diverse sequence space than conventional substitution matrices with 47.3 billion amino acid pairings in 16,295 MSA (as of release 29.0). Additionally, most existing substitution matrices are derived only from highly conserved or filtered sequence data by omitting parts containing gaps or ambiguous amino acids. In contrast, the PFASUM construction takes all information into account. A thorough evaluation of PFASUM’s homology search performance and its capability to produce MSAs shows that this enables PFASUM matrices to significantly outperform commonly used substitution matrices, especially when dealing with sequences of low similarity.

In the following, we will describe the methods for constructing the PFASUM matrices in detail as well as thoroughly discuss their capabilities in comparison to frequently used matrices for two common use cases: Homologous sequence search and MSA construction.

## Method

### PFASUM construction

#### Database selection

As discussed in the introduction, commonly used substitution matrices are derived from quite old and incomplete (filtered) datasets. It is also known that larger and more diverse sequence datasets can produce significantly better performing substitution matrices [25, 35]. Hence, we chose the Pfam seed alignment dataset [16] as basis for our PFASUM substitution matrices. This dataset consists of numerous MSAs (16,295 MSAs in Pfam release 29.0 [16]) covering the currently known sequence space. Each MSA contains a set of representative sequences for a specific group of proteins such as a protein family or domain. This allows our algorithm to capture substitution events between closely related sequences. Combining all MSAs, and thus different groups of sequences, into a single matrix enables us to apply derived substitution events on protein sequences with distant relationships. Furthermore, all Pfam MSAs are manually curated by experts and thus represent ground truth structural alignments.

#### PFASUM algorithm

Substitution matrices usually represent substitution rates in form of rounded log-odds scores derived from aligned and filtered sequence data. For two different amino acids *α*_*i*_ and *α*_*j*_ the unrounded score $S_{\alpha _{i},\alpha _{j}}$ corresponds to $S_{\alpha _{i},\alpha _{j}} = \log _{2} p(\alpha _{i},\alpha _{j}) - \log _{2}\left (p(\alpha _{i}) p(\alpha _{j})\right)$. The term *p*(*α*_*i*_,*α*_*j*_) denotes the substitution frequencies for *α*_*i*_ and *α*_*j*_ which are derived by counting all *α*_*i*_ *α*_*j*_ pairings *n*(*α*_*i*_,*α*_*j*_) and relating these to all counted pairs, i.e. $N = {\sum \nolimits }_{\alpha _{i},\alpha _{j}}n(\alpha _{i},\alpha _{j})$. The terms *p*(*α*_*i*_) and *p*(*α*_*j*_) represent the marginals for observing amino acid *α*_*i*_ and *α*_*j*_ respectively. These can be directly derived by summing over the probability of conservation *p*(*α*_*i*_,*α*_*i*_) and all substitution events (${\sum \nolimits }_{j\neq i} p(\alpha _{i},\alpha _{j})$). The resulting real-numbered log-odds score $S_{\alpha _{i},\alpha _{j}}$ is then rounded to the next integer value.

Our PFASUM algorithm and the corresponding matrices also follow this basic principle. We process each MSA in the Pfam seed dataset separately, accumulate the counted substitution frequencies in a single matrix and subsequently transform these to the final rounded log-odds scores. In order to process unfiltered Pfam seed alignments and to handle special cases such as oversampling issues and ambiguous amino acid symbols, it is, however, necessary to introduce additional steps in the matrix generation.

#### Sequence clustering

Counting amino acid substitutions in a set of highly redundant sequences may result in potential bias from oversampling. To mitigate this problem, Henikoff and Henikoff [22] uses a clustering algorithm for the BLOSUM-*t* matrix calculation to group sequences of equal length *λ* depending on their relative similarity *Φ* and a preset clustering threshold *t*.

Instead of counting the observed amino acid substitutions *n*(*α*_*i*_,*α*_*j*_) for each sequence pair *A* and *B* fully, each substitution is counted as *n*(*α*_*i*_,*α*_*j*_)/|*c*_*x*_|∗|*c*_*y*_|. Thereby, *c*_*x*_ corresponds to the cluster that contains sequence *A* and *c*_*y*_ to the cluster containing sequence *B*. The cardinalities |*c*_*x*_| and |*c*_*y*_| represent the corresponding cluster sizes, i.e. the number of sequences within the clusters. If both sequences *A* and *B* belong to the same cluster, i.e. *c*_*x*_=*c*_*y*_, all substitutions between *A* and *B* are ignored in counting pairs. In other words, a single cluster is considered as a single sequence in counting pairs.

First, the clustering algorithm calculates the similarity *Φ*(**A**,**B**) between two sequences *A* and *B*. The similarity *Φ*(**A**,**B**) between two sequences *A* and *B* is measured by counting the number of aligned positions that share the same amino acid type, normalized by the length *λ* of both sequences. The similarity value is then compared against the preset clustering threshold *t* to decide whether the sequences should be grouped or not. For example, if sequences *A* and *B* are *Φ*(**A**,**B**)=73.5*%* identical and the clustering threshold is set to *t*=62, the sequences are grouped within a cluster. Additional sequences *C* are assigned to this cluster if there exists at least one sequence *X* inside the cluster that is at least *t* % similar to *C*, i.e. *Φ*(**C**,**X**)≥*t*.

The PFASUM algorithm incorporates this method to mitigate oversampling problems. Analogous to the BLOSUM matrices, the number suffix in a matrix’ name, e.g. PFASUM43, indicates the chosen clustering threshold used for the construction of the matrix. However, we had to adapt the similarity measure to cope with the aligned sequences found in the Pfam seed dataset as explained in the following section.

#### Sequence similarity

Two aligned sequences *A* and *B* from a Pfam seed alignment can be directly compared as both can be considered as correctly aligned. A gap symbol found in these alignments is denoted as *γ*. We define **A** as a vector of amino acid and gap symbols with the length *L*, i.e. **A**=(*α*_1_,…,*α*_*L*_). The number of amino acid symbols in **A**, i.e without the gap symbol *γ*, is denoted as *λ*_*A*_. Similarly, **B** is defined as (*β*_1_,…,*β*_*L*_) and *λ*_*B*_ represents the number of amino acids in **B**. The unnormalized similarity *ϕ* between **A** and **B** is defined as:

1 $$ \phi(\mathbf{A},\mathbf{B}) = \sum\limits_{l = 1}^{L} \delta(\alpha_{l},\beta_{l}) \left[1-\delta(\alpha_{l},\gamma)\right] \left[1-\delta(\beta_{l},\gamma)\right]  $$

Hereby, *δ*(*x,y*) is the Kronecker delta which equals one if *x*=*y*, and zero otherwise. In other words, we omit all pairings that contain at least one gap symbol and count only pairs with identical one letter codes. The fractional similarity value *Φ* is computed by normalizing *ϕ*(**A**,**B**) with the length of the shorter sequence, i.e. min(*λ*_*A*_,*λ*_*B*_).

#### Group size normalization

Generally, Pfam alignments represent groups of related protein regions. For our purpose, these groups of sequences are collections of related proteins. Within the broad term “group” we encapsulate protein families, domains, and similar organizational structures. Substitution matrices derived from Pfam seed alignments aim at capturing the average evolutionary behavior, while the number of sequences within each group can vary widely. To avoid over-representing groups with high sequence counts, the PFASUM algorithm derives group-specific pair frequency counts *p*_*k*_(*α*_*i*_,*α*_*j*_) for each sequence group *k*. These *p*_*k*_(*α*_*i*_,*α*_*j*_) are obtained by normalizing the pair counts *n*_*k*_(*α*_*i*_,*α*_*j*_) found in group *k* with the number of sequences *s*_*k*_ in this group. Pair frequencies *p*(*α*_*i*_,*α*_*j*_) for the entire database are then obtained by summing over all normalized *p*_*k*_(*α*_*i*_,*α*_*j*_).

#### Gaps

The Pfam seed dataset contains complete MSAs and thus gaps occur frequently to compensate for different sequence lengths induced by deletions and insertions of amino acids. This is contrary to the data basis of most conventional substitution matrices, e.g. the BLOCKS database used for the BLOSUM construction [21]. These datasets are usually filtered by omitting alignment parts containing gaps. Rather than neglecting MSA columns with at least one gap, the PFASUM algorithm simply neglects gap/amino acid (as well as gap/gap) pairings in counting substitution frequencies. Hence, the PFASUM algorithm considers all found amino acid pairings even in gap-rich columns with few amino acids. This allows us to extract unique information about substitution events even within insertion/deletion regions (indels) since these regions are also manually curated and thus can be considered as reliably aligned.

#### Ambiguous amino acids

Ambiguous amino acid characters – such as B, J, Z and X – occur rarely in most sequence databases, especially in older databases. This consequently results in very low frequencies for pairs that involve ambiguous amino acids so that the computed relative pair frequencies often vanish. Hence, most substitution matrix algorithms fully ignore observed ambiguous amino acids when counting pair frequencies. Instead, matrix entries for these characters are subsequently generated from averaging the pair frequencies of the canonic amino acids, following the translation scheme shown in Table 1. The number of ambiguous amino acids can be, however, larger in modern sequence databases such as Pfam and thus can have a greater influence on the observed substitution frequencies.

**Table 1:** Ambiguous amino acids and their designated canonic amino acids shown as one letter codes

Ambiguous amino acid	B	J	Z	X
Canonic amino acid	N, D	E, Q	I, L	all

To correctly account for this, PFASUM fully processes ambiguous amino acids in counting substitution frequencies. As ambiguous amino acids encode at least two canonic amino acids, it is necessary to redefine amino acids as a set *Θ*_*x*_ of symbols with *x* representing the one letter code of the amino acid. Canonic amino acids are thus represented as sets with a cardinality of one, e.g. *Θ*_*A*_={*A*}. Ambiguous amino acids are defined as sets containing their encoded canonic acids, e.g. *Θ*_*B*_={*N,D*}.

The PFASUM algorithm distributes pair counts *Θ*_*x*_ *Θ*_*y*_ of any found ambiguous amino acid equally among their canonic amino acids, again following Table 1. Also, we count the observed *Θ*_*x*_ *Θ*_*y*_ directly. For example, each found amino acid substitution *Θ*_*A*_ *Θ*_*B*_ in group *k* is counted as 0.5 *Θ*_*A*_ *Θ*_*N*_, 0.5 *Θ*_*A*_ *Θ*_*D*_, and 1.0 *Θ*_*A*_ *Θ*_*B*_.

Afterwards, the final pair frequency counts for all acid-to-acid combinations *x,y* are obtained using the following formula which accounts for counting *Θ*_*x*_ *Θ*_*y*_ more than once if an ambiguous amino acid is involved:

2 $$ \begin{aligned} \mu_{k}\left(\Theta_{x},\Theta_{y}\right) &=\frac{\left[\sum\limits_{\alpha_{i} \in \Theta_{x}}\sum\limits_{\alpha_{j} \in \Theta_{y}}n_{k}\left(\alpha_{i},\alpha_{j}\right)\right] - n_{k}\left(\Theta_{x},\Theta_{y}\right)} {|\Theta_{x}||\Theta_{y}|}\\ p_{k}\left(\Theta_{x},\Theta_{y}\right) &= \frac{\mu_{k}\left(\Theta_{x},\Theta_{y}\right) + n_{k}\left(\Theta_{x},\Theta_{y}\right)} {N_{k}} \end{aligned}  $$

In *p*_*k*_, we add a correction term *μ*_*k*_(*Θ*_*x*_,*Θ*_*y*_) to the number of observed amino acid pairings *n*_*k*_(*Θ*_*x*_,*Θ*_*y*_). This term equates to zero for pairings of canonic amino acids. For pairings involving ambiguous amino acids, *μ* copes with adding these pairings to both canonic amino acids pair counts as well as ambiguous acid pair counts. This ensures that each observed amino acid pairing is only counted once. The normalization factor *N*_*k*_ corresponds to the total number of observed amino acid pairings in group *k*.

Using the example from above, a single observed AB substitution would result in frequency counts *n*_*k*_(*Θ*_*A*_,*Θ*_*N*_)=*n*_*k*_(*Θ*_*A*_,*Θ*_*D*_)=0.5 and *n*_*k*_(*A,B*)=1. The resulting relative frequencies using the formula above yield then *p*_*k*_(*Θ*_*A*_,*Θ*_*N*_)=*p*_*k*_(*Θ*_*A*_,*Θ*_*D*_)=0.5 and *p*_*k*_(*Θ*_*A*_,*Θ*_*B*_)=1.

### Evaluation of homology search performance

One of the most common tasks employing substitution matrices is the identification of homologous sequences. We thus evaluated the capabilities of PFASUM matrices for this particular task in two different scenarios. First, we compare the performance of commonly used substitution matrices such as BLOSUM62 with PFASUM matrices of similar relative entropy. This scenario represents the state-of-the-art approach for comparing substitution matrices as per Altschul [2] on basis of their general compositional properties. Secondly, we investigate homology search performance in a more user centered way by analyzing which of the tested matrices performed best on different databases, while ignoring their relative entropies. The following sections describe the different matrix test sets and databases used for this evaluation as well as the underlying methodology in detail.

#### Tested substitution matrices

We calculated PFASUM matrices using integer clustering thresholds ranging from 0 to 100. Depending on the input data, too small clustering thresholds can lead to clustering results only containing a single large “super-cluster”. Since amino acid substitutions are only counted between different clusters and not within the clusters, this can result in matrices that do not report substitution rates for all possible amino acid substitutions. Hence, we omitted all PFASUM matrices with clustering thresholds <10. We denote the finally obtained matrix set in the following as *PFASUM Search Matrices* (Table 2).

**Table 2:** The matrix test sets assessed in the homology search performance evaluation

Test set	Algorithm	Matrix numbers
*PFASUM Search Matrices*	PFASUM	[11,100]
*Standard Search Matrices*	BLOSUM	50,62,80
	MD	10,20,40
	Optima	5
	PAM	120,250
	VTML	10,20,40,80,120,160,200

In order to evaluate the homology search performance of *PFASUM Search Matrices* against state-of-the-art substitution matrices, we focus on a set of commonly used substitution matrices denoted as *Standard Search Matrices* (Table 2). This set contains various BLOSUM, MD, Optima, PAM, and VTML matrices which are used, e.g., as default parameter in popular homology search tools such as SSEARCH/FASTA [33] and BLAST [3].

#### Databases

To investigate the performance differences between *PFASUM Search Matrices* and *Standard Search Matrices* we conducted homology search experiments on basis of the most recent ASTRAL 2.06 [8, 9] datasets, a subset of sequences of the SCOP/SCOPe database [17, 32]. The ASTRAL database, and especially its ASTRAL40 subset containing sequences with a maximum similarity of 40%, has been commonly suggested as the gold standard for homology search performance evaluation [7, 20, 35, 41]. In order to evaluate substitution matrix performance for different application purposes, we differentiated between three distinct test scenarios and thus ASTRAL database subsets.

While the ASTRAL40 subset represents the state-of-the-art homology search benchmark, the ASTRAL70 dataset emulates database searches against very similar and closely related sequences. We also conducted performance evaluation on basis of the ASTRAL20 dataset, which simulates homology searches of novel proteins with unknown structural features and few known homologs. Effectively, these three datasets allow us to evaluate the performance of substitution matrices for different evolutionary distances.

#### Search methods

In order to obtain the most accurate results for the evaluation of homology search performance we employed the SSEARCH [33] algorithm of FASTA (Version: 36.3.8d). SSEARCH was reported previously to possess higher accuracy than BLAST [20, 22, 41]. In order to avoid potential bias introduced through inaccurate gap parameter settings, we varied gap opening and gap extension penalties from −5 to −20 and −1 to −3, respectively. For each gap parameter and substitution matrix combination, we generated a list of found potential homologous relations when searching all sequences of an ASTRAL dataset to the entire database. These relations were ordered based on their *E*-values, i.e. the probability of obtaining a hit for an unrelated sequence with equal length by pure chance.

#### Performance evaluation

We evaluated the homology search performance of the tested substitution matrices using the coverage measure $\boldsymbol{\mathcal {Q}}$ [7]. For a list of homologous search results ordered by their *E*-values, this measure represents the fraction of the correctly found, true positive superfamily relations which remain after cutting the list in order to restrict the number of false positives to a certain amount. We set this threshold to 0.01 errors per query (epq) in concordance to other studies [25, 35]. This effectively restricts the number of false positives found within 100 queries to a single false positive. To reduce the bias introduced by different superfamily sizes, we used the quadratically normalized version of the coverage as suggested by Price et al. [35]:

3 $$ \mathcal{Q} = \frac{1}{S}\sum\limits_{i=1}^{S} \frac{t_{\mathrm{i}}}{\left(s_{i}^{2} - s_{i}\right)}  $$

*S* represents the number of superfamilies and *t*_*i*_ the number of true positive relations in superfamily *i* which contains *s*_*i*_ sequences.

In order to estimate the significance of the calculated coverages $\boldsymbol{\mathcal {Q}}$ and the differences between them, we used a Concerted Bayesian bootstrapping approach to estimate variance and mean of the underlying coverage distribution [20, 35]. Through variation of the prior distribution, this allows us to evaluate changes in the database composition and accompanying variation in coverage. We obtained sequence weights of the prior from the Dirichlet distribution and conducted the quadratic normalization of the resulting bootstraps as described in Hess et al. [25]. Prior distributions were generated 500 times.

The significance of the coverage differences were measured through *Z*-score statistics which express the significance of the difference between distribution *M* and *P* as:

4 $$ Z_{M,P} = \frac{\mathcal{\bar{Q}}_{M} - \mathcal{\bar{Q}}_{P}}{\sqrt{\frac{\sigma^{2}_{M} + \sigma^{2}_{P}}{N}}}   $$

Here, $\mathcal {\bar {Q}}_{M}$ represents the mean coverage for distribution *M* with its corresponding variance $\sigma ^{2}_{M}$ and *N* the number of bootstrap steps, i.e. 500 in our case.

### Evaluation of MSA construction

Another popular task for employing substitution matrices is the construction of Multiple Sequence Alignments (MSA). Hence, we also evaluated the impact of PFASUM matrices on the quality of MSAs using state-of-the-art MSA benchmarks. The following sections describe this evaluation in detail.

#### Tested substitution matrices

The calculation of pairwise sequence alignments forms the basis of many homology search tools and MSA programs. For example, the search tools SSEARCH [33] and BLAST [3] employ pairwise alignments for calculating the similarity between sequences. MSA programs such as MUSCLE [14] and MAFFT [28] use pairwise alignments, e.g., during guide tree construction or when generating profile-profile alignments. This suggests that matrices which are suitable for either task may also be useful for the other task. Hence, we assess PFASUM’s MSA construction capabilities by focusing on the three best performing *PFASUM Search Matrices* in our homology search performance evaluation, namely PFASUM31, PFASUM43 and PFASUM60 (Additional file 2: Figure S2; Additional file 3: Figure S3; Additional file 4: Figure S4). We refer to this matrix subset in the following as *PFASUM MSA Matrices* (Table 3).

**Table 3:** The matrix test sets assessed in the MSA construction evaluation

Test set	Algorithm	Matrix numbers
*PFASUM MSA Matrices*	PFASUM	31,43,60
*Standard MSA Matrices*	BLOSUM	50,62
	PAM	250
	VTML	160,200

Out of the set of *Standard Search Matrices*, we chose the PAM250, BLOSUM50, BLOSUM62, VMTL160, and VTML200 matrices for this evaluation. These matrices are used as default matrix by several MSA algorithm such as MUSCLE and MAFFT. We refer to this matrix subset in the following as *Standard MSA Matrices* (Table 3).

#### Benchmark datasets

To compare the quality of MSAs generated using our novel PFASUM matrices with those created with conventional matrices, we used the MSA benchmark collection bench provided by R.C. Edgar [12]. This collection of benchmark datasets consists of commonly used MSA benchmarks stored in FASTA [34] format. From these, we selected the unmodified BAliBASE 3.0 [42], SABmark 1.65 [43] and OXBench [36] benchmarks for our evaluation. Each benchmark consists of reference MSAs and corresponding unaligned sequence sets.

BAliBASE 3.0 is one of the most widely used MSA benchmarks and provides 386 MSAs categorized in five different sets. Each set represents a specific MSA use case, e.g. a set of very divergent sequences (Reference 1) or sequence families that are aligned to a distantly related sequence (Reference 2). The MSAs in each set were generated using a combination of sequence- and structure-based methods with manual refinement [15]. SABmark 1.65 provides two sets of MSAs, a “Twighlight Zone” set (209 MSAs) and a “Superfamilies” set (425 MSAs) which are derived from a consensus of SOFI and CE [6]. While the sequences in the first set share a maximum similarity of 25%, the second set contains sequences with a maximum similarity of 50%. The underlying sequences were selected using fold information from the SCOP database and thus possess known structure. The last benchmark used in our evaluation is OXBench. It provides a set of 395 structural MSAs constructed using STAMP [38] and 3D structural information from the 3Dee database [39].

#### MSA methods

For our evaluation, we constructed 543,360 MSAs in total using the popular MUSCLE algorithm (v3.8.425) [14] in combination with the aforementioned substitution matrices and different gap penalties. Similar to our homology search performance evaluation, we varied the gap opening and gap extension penalties between −5 and −20 and −1 and −3, respectively, to prevent the bias from potentially inaccurate gap penalty settings.

MUSCLE constructs MSAs in three steps, i.e. a draft progressive, an improved progressive and an iterative refinement step. In order to mitigate MSA quality differences solely induced by refinement steps, we set the maximum number of iterations to one. The MSAs are thus computed using a matrix independent guide tree and a single progressive alignment step only based on our chosen parameters. Hence, the quality of the generated MSAs only depends on the evaluated substitution matrix and gap penalties.

#### MSA quality evaluation

We measured the quality differences between our generated MSAs and the reference MSAs using the *q*-score measure [14] implemented in the identically named tool qscore by R.C. Edgar [13]. This measure describes the fraction of identically and thus correctly aligned amino acid pairs between a test and a reference MSA. In other words, the quality of a test MSA can be expressed as a number between 0 and 1.

We use the *q*-score in two different evaluation scenarios. First, we calculate the average *q*-score $\bar {q}$ over all MSAs in a benchmark dataset for each substitution matrix separately. This allows a general comparison but is obviously sensitive to strong outliers. To compensate this issue, we provide a second evaluation scenario. Here, we count the number of times that a specific PFASUM matrix in the *PFASUM MSA Matrices* set produced an MSA of at least as good quality as a specific matrix out of the *Standard MSA Matrices* set.

## Results

In the following we will first show the results for the performance of matrices in the *PFASUM Search Matrices* and *Standard Search Matrices* sets for homologous sequence search (Table 2). The performance of these will be evaluated on the *de facto* gold standard for homologous sequence search, the ASTRAL database. In the second part of this section, we will examine the MSA generation capabilities of PFASUM matrices compared to conventional matrices using the *PFASUM MSA Matrices* and *Standard MSA Matrices* sets. Here, we investigate three distinct scenarios for computing MSAs from different sequence compositions using the benchmark datasets BAliBASE 3.0, OXBench and SABmark 1.65.

### Homologous sequence search

In order to gauge the performance of PFASUM substitution matrices in the context of homologous sequence search, we first present results for matrices with comparable relative entropies. As the relative entropy describes the divergence of observed substitution events and independent evolution captured within a substitution matrix, a fair performance comparison between matrices can only be achieved if they possess similar relative entropies [2]. In the second part, we present the results of our general and entropy-independent performance evaluation. While the former part evaluates general compositional properties of substitution matrices (and to some extend the underlying algorithm), the latter part highlights which substitution matrices performed best in our test settings.

#### Comparison based on relative entropy

In order to properly assess the performance of substitution matrices, Altschul [2] suggested comparing matrices with similar relative entropy. The entropy represents the information divergence between independent and observed evolutionary relations. The relative entropy for *PFASUM Search Matrices* ranges between 0.0668 bits (PFASUM11) and 0.7319 bits (PFASUM100). *Standard Search Matrices* such as VTML10, VTML20, VTML40, VTML80, PAM120 and BLOSUM80 thus cannot be directly compared to *PFASUM Search Matrices*, since their entropies differ drastically from the *PFASUM Search Matrices* entropy range (Additional file 5: Table S1).

**Fig. 1:**
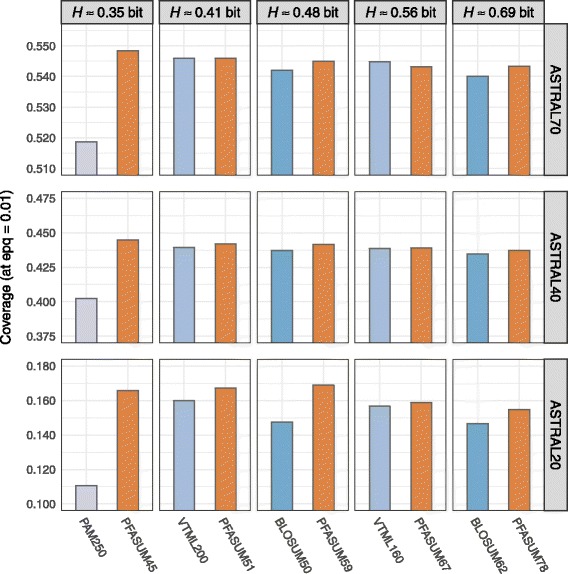
Performance comparison of *Standard Search Matrices* with *PFASUM Search Matrices* of similar entropies on all three ASTRAL datasets. The highest achieved coverage at an 0.01 errors per query for any gap opening and extension penalty combination is shown. Best performing gap parameter for each matrix and database combination can be found in Additional file 6: Table S2. Please note, we reduced the shown range of the coverage to emphasize the differences between the matrices

For all other matrices, we compared their performance on the three ASTRAL datasets for varying gap penalty settings. Figure 1 shows the highest achieved coverages at 0.01 errors per query (epq) for *Standard Search Matrices* and their comparable PFASUM counterparts each using individual best performing gap penalties (Additional file 6: Table S2). The significance of the results was estimated with *Z*-score statistics using Concerted Bayesian bootstrap (Additional file 7: Table S3).

The obtained coverage results and *Z*-Scores show that *PFASUM Search Matrices* always perform at least as good or significantly better than their comparable *Standard Search Matrices* with one single exception: The VTML160 matrix performs slightly better on the ASTRAL70 dataset. This performance difference can be related to the different matrix compositions. While the diagonal entries of VTML160 and its counterpart PFASUM67 are very similar, there are numerous differences of up to four log-odds scores in PFASUM67 when comparing the off-diagonal entries. PFASUM67 thus favors more substitution events than VTML160 which may be useful when searching for remote homologs. For datasets containing similar sequences such as ASTRAL70, however, this can result in more false positive relationships identified.

On a global level, the performance advantage of *PFASUM Search Matrices* over the tested *Standard Search Matrices* grows with decreasing sequence similarity in the test databases. While the performance differences on ASTRAL70 and ASTRAL40 are only marginal but still significant, the coverage differences for ASTRAL20 are much greater. This indicates that *PFASUM Search Matrices* are especially useful for detecting remote homologs.

#### Entropy-independent search performance comparison

Henikoff and Henikoff [23] showed that substitution matrices of a given matrix family perform best around a relative entropy *H* of ∼ 0.7 bit. We chose to re-evaluate this hypothesis on basis of the Pfam seed database. Similar to Hess et al. [25], our results show that the best performing substitution matrices – including *PFASUM Search Matrices* – possess relative entropies well below the suggested 0.7 bit.

**Fig. 2:**
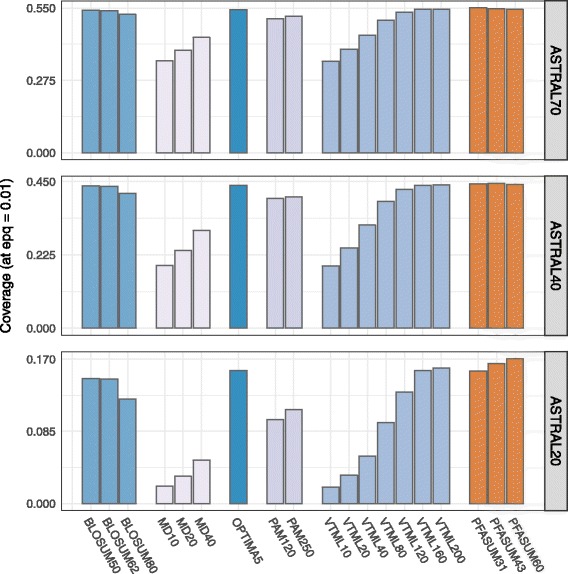
Comparison of the performance of all *Standard Search Matrices* with the novel *PFASUM Search Matrices* on three different ASTRAL datasets. The highest achieved coverage at 0.01 errors per query for any gap opening and extension penalty combination is shown (parameters are listed in Additional file 8: Table S4). With the exception of two performance differences, all shown coverage values are significantly different according to our Z-score analysis shown in Additional file 9: Table S5

Figure 2, shows the performance comparison of all Standard Search Matrices with the three best-performing PFASUM Search Matrices on the tested ASTRAL datasets. The values represent the highest achieved coverage at 0.01 errors per query for any gap opening and extension penalty combination (parameters are listed in Additional file 8: Table S4). The best performing matrices on the datasets ASTRAL40 and ASTRAL70 are PFASUM43 (*H*= 0.3354 bit, Additional file 3: Figure S3) and PFASUM31 (*H*= 0.2297 bit, Additional file 2: Figure S2).

We also find that PFASUM matrices with higher matrix number perform better on sequence data with lower sequence similarity than matrices with lower matrix number (Additional file 1: Figure S1). Whereas PFASUM60 shows the best performance on the ASTRAL20 dataset, the best performing PFASUM matrices for sequences with relatively high sequence similarity can be found at low clustering thresholds with PFASUM31 outperforming all others on the ASTRAL70 dataset.

When comparing the three top performing *PFASUM Search Matrices* to all *Standard Search Matrices*, we find that *PFASUM Search Matrices* deliver superior homology search performance with significantly greater coverage values (Fig. 2) as indicated by the corresponding Z-scores (Additional file 9: Table S5). The highest improvements in coverage over *Standard MSA Matrices* were achieved on the ASTRAL20 dataset. Similar to our findings in the evaluation based on similar entropy, this indicates that *PFASUM Search Matrices* are especially useful when searching for remote homologs. Surprisingly, the often used BLOSUM matrices are outperformed by VTML200 and the OPTIMA5 matrix. Additionally, BLOSUM80 – often suggested as matrix of choice for sequence data sets with high similarity – is outperformed by BLOSUM50 and BLOSUM62 on all three test datasets. Both PAM matrices exhibit relative good performance on the high similarity dataset (ASTRAL70), but either is under-performing for sequences with more remote evolutionary relation (ASTRAL20). The MD [26] matrices deliver similar results to the lower numbered VTML matrices.

While VTML200 tends to be an universally good choice for homology search as the best performing matrix out of the *Standard Search Matrices* set on all three datasets, *PFASUM Search Matrices* can still achieve higher coverage values (Table 4). In general, the best performing *PFASUM Search Matrices* for the ASTRAL20, ASTRAL40 and ASTRAL70 datasets outperform all *Standard Search Matrices* on a statistical significant level (Additional file 9: Table S5).

**Table 4:** Best performing substitution matrices of the *PFASUM Search Matrices* and *Standard Search Matrices* sets for the three test scenarios

Database	Matrix	Gap parameters	Coverage
ASTRAL20	VTML200	-14/-1	0.1598
	PFASUM60	-16/-1	0.1706
ASTRAL40	VTML200	-14/-1	0.4392
	PFASUM43	-13/-1	0.4448
ASTRAL70	VTML200	-9/-2	0.5459
	PFASUM31	-13/-2	0.5508

### Multiple sequence alignments

Whereas homologous sequence search assessment aims at evaluating the performance of substitution matrices for pairwise sequence alignments, the alignment of multiple sequences in MSAs is another field of application for substitution matrices. We will first compare the average performance of matrices in the *PFASUM MSA Matrices* set to conventional substitution matrices grouped in the *Standard MSA Matrices* set (Table 3) on three popular MSA benchmark datasets. In the second part, we will dissect these results and investigate how *PFASUM MSA Matrices* fare on single MSAs in comparison to *Standard MSA Matrices*.

#### Average matrix performance

**Fig. 3:**
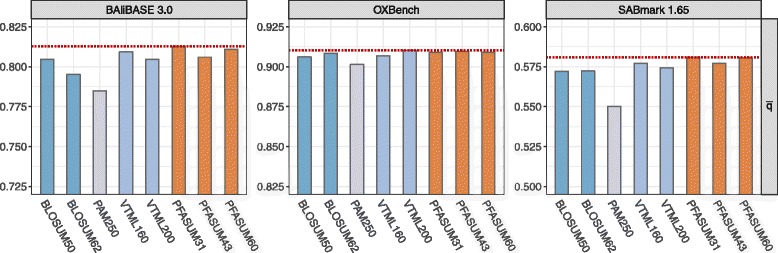
General comparison of MSA matrix performance based on the average *q*-score $\bar {q}$ per benchmark database. *PFASUM MSA Matrices* outperform the tested *Standard MSA Matrices* on all three benchmarks. PFASUM31 achieved the highest $\bar {q}$ for BAliBASE 3.0 and SABmark 1.65, while VTML200 leads all matrices on the OXBench dataset. The red dotted line indicates the maximum $\bar {q}$ separately for each benchmark

To properly evaluate the capabilities of *PFASUM MSA Matrices* in comparison to *Standard MSA Matrices* for MSA construction, we computed MSAs based on three different MSA benchmark datasets using the MSA program MUSCLE in combination with the aforementioned matrices and varying gap penalties. The quality differences between these MSAs and their benchmark reference MSAs were measured afterwards using the *q*-score measure. Figure 3 shows the results for the average *q*-score ($\bar {q}$) for all tested matrices on the BAliBASE, OXBench and SABmark datasets.

As expected, we observed a significantly higher $\bar {q}$ for all matrices on the OXBench dataset than on BAliBASE 3.0 and SABmark since ≥73*%* of the MSAs in this dataset consist of sequences with at least 40% sequence identity. Contrarily, the SABmark dataset presents a decisively more difficult challenge for all matrices with ≥93*%* of all alignments possessing less than 40% sequence similarity. The BAliBASE 3.0 dataset can be placed in between SABmark and OXBench in terms of sequence identity with ≥63*%* at less then 40% similarity.

Even though we analyze the alignment quality of substantially different test datasets, we find our *PFASUM MSA Matrices* in the top three performing matrices on all datasets. On BAliBASE 3.0, PFASUM31 achieved the highest $\bar {q}$ of all matrices with $\bar {q}_{\text {PFASUM31}} = 0.8128$, closely followed by PFASUM60 with $\bar {q}_{\text {PFASUM60}} = 0.8110$ and VTML160 with $\bar {q}_{\text {VTML160}} = 0.8093$. The highest alignment quality on OXBench is reported for VTML200 (at $\bar {q}_{\text {VTML200}} = 0.9102$) only marginally besting PFASUM60 with $\bar {q}_{\text {PFASUM60}} = 0.9095$. For the SABmark dataset we find the highest two performances for PFASUM31 ($\bar {q} = 0.5808$) and PFASUM60 ($\bar {q}= 0.5804$). In this case, the next highest average alignment quality by a matrix out of the *Standard MSA Matrices* set was achieved by VTML160 ($\bar {q}= 0.5771$).

The SABmark dataset allows us to delve deeper in the performance of substitution matrices on alignments with very low sequence identity (’twilight zone’ dataset) and moderately difficult alignments (superfamily dataset). On both datasets, we observe that *PFASUM MSA Matrices* outperform the *Standard MSA Matrices* with PFASUM60 achieving the highest $\bar {q}$ for the ’twilight zone’ dataset, while PFASUM31 performed the best for the superfamily alignments (Additional file 10: Table S6).

In summary, *PFASUM MSA Matrices* outperform all analyzed *Standard MSA Matrices* on average on benchmark datasets with low sequence identity with PFASUM60 being the matrix of choice for very difficult alignments of sequences with low sequence identity. For alignments with moderate complexity and medium sequence similarity, PFASUM31 proofs to generate better alignments than any of the *Standard MSA Matrices*.

#### Quality improvements over *Standard MSA Matrices*

While the average *q*-score is an overall assessment of the alignment quality, directly comparing the performance between two matrices on alignments can yield insights on whether the average is dominated by strong outliers. Hence, we chose to compare *PFASUM MSA Matrices* with *Standard MSA Matrices* on a per alignment level based on their reported *q*-score values. For this, we count the number of times that a specific tested PFASUM matrix produced an MSA of at least as good or higher quality as a specific matrix out of the *Standard MSA Matrices* set. The results for this comparison on basis of BAliBASE 3.0, OXBench and SABmark are shown in percent in Table 5.

**Table 5:** Fraction of times (in percent) that a specific matrix in the *PFASUM MSA Matrices* set produced an MSA of at least as good (≥) quality as a specific matrix out of the *Standard MSA Matrices* set

		PFASUM31	PFASUM43	PFASUM60
BAliBASE 3.0	BLOSUM50	67.36 (59.07)	62.69 (54.92)	62.44 (51.81)
	BLOSUM62	71.50 (65.03)	69.43 (62.44)	67.88 (58.03)
	PAM250	75.39 (70.21)	71.76 (63.73)	70.73 (66.84)
	VTML160	63.47 (54.92)	61.14 (50.52)	61.66 (48.45)
	VTML200	68.13 (57.77)	63.21 (51.81)	61.92 (50.00)
OXBench	BLOSUM50	81.52 (25.06)	83.29 (26.84)	84.30 (23.80)
	BLOSUM62	79.24 (23.04)	81.01 (22.03)	80.76 (21.52)
	PAM250	80.00 (32.41)	82.03 (31.65)	79.49 (33.16)
	VTML160	79.24 (24.81)	83.80 (28.61)	82.53 (25.32)
	VTML200	76.96 (20.76)	82.03 (22.53)	80.00 (20.51)
SABmark 1.65	BLOSUM50	67.85 (47.52)	63.36 (44.21)	65.96 (43.74)
	BLOSUM62	63.59 (48.23)	64.30 (47.28)	64.30 (45.86)
	PAM250	72.58 (59.57)	70.21 (56.97)	72.81 (60.05)
	VTML160	64.78 (45.39)	60.76 (43.50)	65.25 (42.08)
	VTML200	66.67 (47.99)	63.83 (44.21)	68.56 (44.21)

The comparison for better-than-relations (>) are shown in brackets. Values are shown for all *PFASUM MSA Matrices* vs. *Standard MSA Matrices* comparisons on all three different benchmark datasets

*PFASUM MSA Matrices* achieved a *q*-score at least as good as the *Standard MSA Matrices* in over 60% of all BAliBASE 3.0 alignments, outperforming them in at least 50% of the test cases.

In comparison to PAM250, the usage of *PFASUM MSA Matrices* even resulted in higher quality in over 63% of the test cases. Similar to BAliBASE 3.0, over 60% of all SABmark alignments reconstructed using *PFASUM MSA Matrices* show a comparable quality than those generated with *Standard MSA Matrices* and at least 42% are of higher quality.

In contrast to the other two benchmarks, the performance gain of *PFASUM MSA Matrices* over *Standard MSA Matrices* on OXBench MSAs is rather small. Only 20 to 33% of the MSAs generated with *PFASUM MSA Matrices* show larger q-scores than those constructed with *Standard MSA Matrices*. However, between 76% and 84% of the PFASUM generated MSAs are at least as good as their counterparts. Interestingly, while VTML200 achieves a higher average *q*-score $\bar {q}$ than any of the *PFASUM MSA Matrices* on OXBench alignments, *PFASUM MSA Matrices* still produced higher or equal quality MSAs than VTML200 in over 76% of these alignments.

## Discussion

*PFASUM Search Matrices* perform significantly better than *Standard Search Matrices* in homologous sequence search, especially on datasets with small or limited sequence similarity such as ASTRAL20. The best performing matrix on this dataset is the PFASUM60 matrix with a relative entropy of *H*= 0.4941 bit. Interestingly, this matrix performs slightly worse on more similar datasets such as ASTRAL40 and ASTRAL70 compared to PFASUM43 and PFASUM31 which have much lower relative entropies of only *H*= 0.3354 bit and *H*= 0.2297 bit. For ungapped alignments matrices with higher relative entropy are usually more suitable for detecting homologs within similar sequences than matrices with lower relative entropies [2]. This is apparently not the case when using PFASUM matrices on the tested ASTRAL datasets.

A possible explanation for these findings can be drawn from the composition of the Pfam seed alignments, the basis for *PFASUM Search Matrices*. Pfam seed alignments consist of representative sequences for each family that are aligned based on their structural properties. The sequences within a family are thus structurally similar but do not necessarily possess a similar amino acid composition. When clustering these potentially dissimilar sequences using low clustering thresholds, substitution events between them are thus attenuated. *PFASUM Search Matrices* with lower matrix number, i.e. lower clustering thresholds, thus apparently favor pairs of identical amino acids over substitution events and are more suited for similar sequence datasets despite their relative entropy being small. A full assessment of this effect requires a deep and thorough analysis of the amino acid compositions in the ASTRAL dataset and Pfam seed sequences. This is, however, beyond the scope of this article and recommended for future research.

Our performance evaluation shows that SSEARCH using *PFASUM Search Matrices* provides significantly better search results and as such also higher quality pairwise sequence alignments. Since these form the basis for many state-of-the-art MSA algorithms such as MUSCLE and MAFFT, we also consequently tested the capabilities of a selection of the aforementioned matrices for MSA construction. Our results indicate that the tested *PFASUM MSA Matrices* perform exceptionally well when aligning sequences with medium to low sequence similarity such as in the BAliBASE 3.0 and SABmark 1.65 benchmarks. However, the performance differences between *PFASUM MSA Matrices* and the best performing *Standard MSA Matrices* on datasets containing similar sequences such as OXBench is rather small.

This effect can be related to compositional similarities between the matrices which in particular affects the alignment of similar sequences. All tested matrices in our MSA evaluation, with the exception of PAM250, share comparable scoring ratios between diagonal and off-diagonal entries per amino acid, favoring amino acid conservation over substitutions. Since similar sequences are usually more conserved and the majority of the matrix differences can be on the off-diagonal, the alignments generated with *PFASUM MSA Matrices* or *Standard MSA Matrices* only show minor differences.

## Conclusion

We presented the novel PFASUM substitution matrices for the accurate detection of homologous protein sequences and for scoring and constructing high quality protein MSAs. Our PFASUM matrices are based on the Pfam seed dataset [16] (version 29.0) which represents the currently known sequence space covering a large variety of related and divergent sequences. The MSAs in this dataset are also manually curated by experts. Hence, the data basis for PFASUM substitution matrices is not only much larger and diverse than those of conventional substitution matrices, but also represents ground truth data instead of automatically generated and thus potentially biased data. In contrast to conventional construction methods, our algorithm can also effectively handle unfiltered MSAs and ambiguous amino acid symbols and thus prevents the loss of potentially important information. An in-depth evaluation showed, that these features enable PFASUM substitution matrices to deliver significantly better homology search results and produce more accurate MSAs than conventional matrices. One of the best performing PFASUM matrix for homologous sequence search is PFASUM60, especially when searching for distantly related homologs. PFASUM60 also showed reasonable quality improvements for MSA construction. We thus recommend PFASUM60 as a general choice for these particular tasks.

## Additional files

Additional file 1 **Figure S1**. Progress of coverage for different PFASUM matrix numbers. Shown are the highest coverage values obtained for the different ASTRAL subsets for a given clustering threshold, regardless of the gap parameter settings. (PDF 37.8 kb)

Additional file 2 **Figure S2**. PFASUM31 matrix (*H*=0.2297 bit) constructed from all Pfam seed alignments (version 29.0) with a 31% sequence similarity threshold. (PDF 41.6 kb)

Additional file 3 **Figure S3**. PFASUM43 matrix (*H*=0.3354 bit) constructed from all Pfam seed alignments (version 29.0) with a 43% sequence similarity threshold. (PDF 41.7 kb)

Additional file 4 **Figure S4**. PFASUM60 matrix (*H*=0.4941 bit) constructed from all Pfam seed alignments (version 29.0) with a 60% sequence similarity threshold. (PDF 41.6 kb)

Additional file 5 **Table S1**. Table of *Standard Search Matrices* with their relative entropy as listed in FASTA (Version: 36.3.8d, found in upam.h). Comparable PFASUM substitution matrices are listed with their respective entropy. Note, we list entries with n/a where no comparable PFASUM matrix can be found due to the distribution of entropies of possible PFASUM matrices. No matrix entropy is listed for OPTIMA5 and MD matrices. (PDF 42.6 kb)

Additional file 6 **Table S2**. Table of gap parameters used for matrices at similar entropy levels that resulted in the highest coverage. (PDF 32.2 kb)

Additional file 7 **Table S3**. Table of Z-score values for the performance comparison of *Standard Search Matrices* with their PFASUM counterparts based on relative entropy. Non-significant Z-scores based on the 95% percentile are highlighted as bold. (PDF 43.0 kb)

Additional file 8 **Table S4**. Table of all substitution matrices in the homology search evaluation showing their highest coverage and their corresponding gap parameters for all three databases. (PDF 43.0 kb)

Additional file 9 **Table S5**. Table of Z-score values for the comparison between *PFASUM Search Matrices* and *Standard Search Matrices* on the three different ASTRAL datasets. Z-scores with |*Z*|≥1.96 represent statistically significant underlying distributions at the 95% confidence interval. Non-significant Z-scores are highlight in red. (PDF 60.6 kb)

Additional file 10
**Table S6**. Average *q*-score $\bar{q}$ for the SABmark alignments, split between superfamily alignments and so-called “twilight zone” alignments. Matrices with the highest performances are highlighted in bold. (PDF 47.5 kb)
