# Supplementary material for: PFASUM: a substitution matrix from Pfam structural alignments
Source: BMC Bioinformatics. 2017 Jun 5;18:293. doi: 10.1186/s12859-017-1703-z (PMC5460430; doi:10.1186/s12859-017-1703-z)
Supplement: Supplementary file 1 — Figure S1. Progress of coverage for different PFASUM matrix numbers. Shown are the highest coverage values obtained for the different ASTRAL subsets for a given clustering threshold, regardless of the gap parameter settings. (PDF 37.8 kb) [file 12859_2017_1703_MOESM1_ESM.pdf]

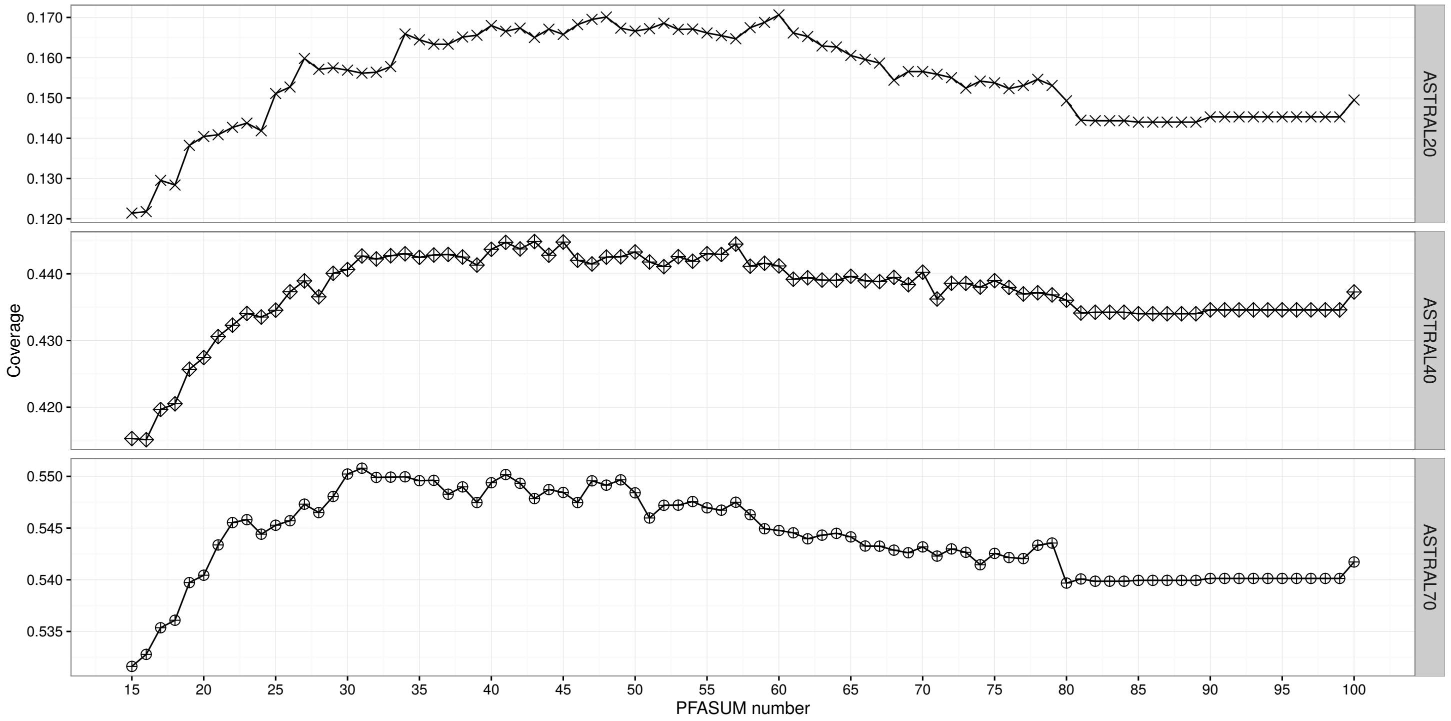

Additional figure 1: Progress of coverage for different PFASUM matrix numbers. Shown are the highest coverage values obtained for the different ASTRAL subsets for a given clustering threshold, regardless of the gap parameter settings.
