# Supplementary material for: PFASUM: a substitution matrix from Pfam structural alignments
Source: BMC Bioinformatics. 2017 Jun 5;18:293. doi: 10.1186/s12859-017-1703-z (PMC5460430; doi:10.1186/s12859-017-1703-z)
Supplement: Supplementary file 2 — Figure S2. PFASUM31 matrix (H=0.2297 bit) constructed from all Pfam seed alignments (version 29.0) with a 31% sequence similarity threshold. (PDF 41.6 kb) [file 12859_2017_1703_MOESM2_ESM.pdf]

	A	R	N	D	C	Q	E	G	H	I	L	K	M	F	P	S	T	W	Y	V	B	Z	J	X
A	4																							
R	-1	7																						
N	-1	0	7																					
D	-1	0	3	8																				
C	1	-3	-3	-4	16																			
Q	0	2	1	1	-3	5																		
E	-1	1	1	3	-4	3	6																	
G	0	-2	1	0	-2	-1	-1	9																
H	-2	1	1	0	-2	1	0	-2	12															
I	-1	-4	-4	-6	0	-3	-4	-4	-4	5														
L	-1	-3	-4	-5	0	-3	-4	-4	-3	3	5													
K	-1	3	1	1	-4	2	2	-1	0	-4	-4	6												
M	0	-2	-3	-4	0	-1	-3	-3	-2	2	3	-2	6											
F	-2	-4	-4	-5	0	-4	-5	-4	-2	2	2	-4	2	7										
P	-1	-1	-1	0	-3	-1	0	-1	-1	-3	-3	0	-3	-3	10									
S	1	0	1	1	0	0	0	1	0	-3	-3	0	-2	-3	0	4								
T	0	0	1	0	-1	0	0	-1	-1	-1	-2	0	-1	-2	-1	2	5							
W	-2	-2	-4	-5	-2	-3	-4	-3	-1	-1	0	-4	0	3	-3	-3	-3	16						
Y	-2	-2	-2	-3	-1	-2	-3	-3	2	-1	0	-2	0	4	-3	-2	-2	4	9					
V	0	-3	-4	-5	1	-3	-4	-3	-3	4	2	-3	1	1	-2	-2	0	-1	-1	5				
B	-5	-4	-1	-1	-7	-3	-2	-4	-3	-9	-9	-3	-7	-9	-4	-3	-4	-8	-7	-8	-1			
Z	-5	-2	-3	-1	-8	-2	-2	-5	-4	-8	-8	-2	-6	-8	-5	-4	-4	-8	-6	-7	-6	-2		
J	-5	-7	-8	-9	-4	-7	-8	-8	-7	-2	-2	-8	-1	-2	-7	-7	-6	-4	-4	-1	-13	-12	-3	
X	-18	-18	-18	-18	-18	-18	-18	-18	-18	-18	-18	-18	-17	-18	-18	-18	-18	-18	-18	-18	-22	-22	-22	-27

Additional figure 2: PFASUM31 matrix ($H = 0.2297$ bit) constructed from all Pfam seed alignments (version 29.0) with a 31% sequence similarity threshold.
