# Supplementary material for: PFASUM: a substitution matrix from Pfam structural alignments
Source: BMC Bioinformatics. 2017 Jun 5;18:293. doi: 10.1186/s12859-017-1703-z (PMC5460430; doi:10.1186/s12859-017-1703-z)
Supplement: Supplementary file 3 — Figure S3. PFASUM43 matrix (H=0.3354 bit) constructed from all Pfam seed alignments (version 29.0) with a 43% sequence similarity threshold. (PDF 41.7 kb) [file 12859_2017_1703_MOESM3_ESM.pdf]

	A	R	N	D	C	Q	E	G	H	I	L	K	M	F	P	S	T	W	Y	V	B	Z	J	X
A	4																							
R	-1	6																						
N	-1	0	6																					
D	-1	0	2	6																				
C	0	-3	-2	-4	13																			
Q	0	2	1	1	-3	5																		
E	-1	1	1	3	-4	2	5																	
G	0	-2	0	0	-2	-1	-1	7																
H	-2	1	1	0	-2	1	0	-2	9															
I	-1	-3	-4	-5	-1	-3	-4	-4	-3	5														
L	-1	-3	-4	-5	-1	-3	-4	-4	-3	2	4													
K	-1	3	1	0	-4	2	2	-1	0	-3	-3	5												
M	0	-2	-2	-4	0	-1	-3	-3	-2	2	2	-2	6											
F	-2	-3	-3	-5	-1	-3	-4	-4	-1	1	2	-4	1	7										
P	-1	-1	-1	0	-3	-1	-1	-1	-1	-3	-3	-1	-3	-3	9									
S	1	0	1	0	0	0	0	0	0	-3	-3	0	-2	-3	0	4								
T	0	0	0	0	-1	0	0	-1	-1	-1	-2	0	-1	-2	-1	2	4							
W	-2	-2	-3	-4	-2	-3	-4	-3	-1	-1	0	-3	0	3	-3	-3	-3	13						
Y	-2	-2	-2	-3	-1	-2	-3	-3	2	-1	0	-2	0	4	-3	-2	-2	3	8					
V	0	-3	-3	-4	0	-2	-3	-3	-3	3	2	-3	1	0	-2	-2	0	-2	-1	4				
B	-4	-3	0	0	-6	-2	-1	-3	-3	-7	-7	-2	-6	-7	-4	-2	-3	-7	-5	-7	0			
Z	-4	-2	-2	-1	-7	-1	-1	-4	-3	-7	-6	-1	-5	-7	-4	-3	-3	-6	-5	-6	-5	-1		
J	-4	-6	-7	-8	-4	-6	-7	-7	-6	-1	-1	-6	-1	-1	-6	-6	-4	-4	-4	-1	-10	-9	-1	
X	-13	-13	-13	-14	-14	-13	-13	-14	-13	-13	-14	-13	-13	-13	-14	-13	-13	-14	-14	-13	-16	-16	-17	-20

Additional figure 3: PFASUM43 matrix ($H = 0.3354$ bit) constructed from all Pfam seed alignments (version 29.0) with a 43% sequence similarity threshold.
