# Supplementary material for: PFASUM: a substitution matrix from Pfam structural alignments
Source: BMC Bioinformatics. 2017 Jun 5;18:293. doi: 10.1186/s12859-017-1703-z (PMC5460430; doi:10.1186/s12859-017-1703-z)
Supplement: Supplementary file 4 — Figure S4. PFASUM60 matrix (H=0.4941 bit) constructed from all Pfam seed alignments (version 29.0) with a 60% sequence similarity threshold. (PDF 41.6 kb) [file 12859_2017_1703_MOESM4_ESM.pdf]

	A	R	N	D	C	Q	E	G	H	I	L	K	M	F	P	S	T	W	Y	V	B	Z	J	X
A	5																							
R	-1	7																						
N	-2	0	7																					
D	-2	-1	2	7																				
C	0	-4	-3	-5	14																			
Q	-1	2	1	1	-4	6																		
E	-1	0	0	3	-5	2	6																	
G	0	-2	0	-1	-2	-2	-2	8																
H	-2	1	1	0	-2	1	0	-2	10															
I	-1	-4	-5	-6	-1	-4	-5	-5	-4	6														
L	-1	-3	-4	-6	-1	-3	-4	-5	-3	3	5													
K	-1	3	1	0	-4	2	1	-2	0	-4	-4	6												
M	-1	-2	-3	-4	-1	-1	-3	-4	-2	2	3	-2	8											
F	-2	-4	-4	-6	-1	-4	-5	-5	-1	1	2	-5	1	8										
P	-1	-2	-1	-1	-4	-1	-1	-2	-2	-4	-4	-1	-4	-4	10									
S	1	-1	1	0	0	0	0	0	-1	-3	-4	0	-2	-3	0	5								
T	0	-1	0	-1	-1	0	-1	-2	-1	-1	-2	0	-1	-3	-1	2	6							
W	-3	-3	-4	-5	-2	-3	-5	-4	-1	-2	-1	-4	-1	3	-4	-4	-3	14						
Y	-3	-2	-2	-4	-1	-2	-3	-4	2	-2	-1	-3	-1	4	-4	-3	-2	3	9					
V	0	-3	-4	-5	0	-3	-4	-4	-3	4	1	-3	1	0	-3	-2	0	-2	-2	5				
B	-5	-4	0	1	-7	-2	-1	-3	-3	-8	-8	-3	-7	-8	-4	-2	-3	-8	-6	-8	1			
Z	-4	-2	-2	-1	-7	0	0	-5	-3	-7	-7	-1	-5	-8	-4	-3	-3	-7	-6	-6	-5	0		
J	-4	-7	-8	-9	-4	-6	-8	-8	-6	0	-1	-7	-1	-2	-7	-7	-5	-4	-4	-1	-11	-10	0	
X	-14	-14	-13	-14	-14	-13	-14	-14	-13	-14	-14	-13	-13	-14	-14	-13	-13	-14	-14	-14	-17	-16	-17	-21

Additional figure 4: PFASUM60 matrix ($H = 0.4941$ bit) constructed from all Pfam seed alignments (version 29.0) with a 60% sequence similarity threshold.
