# Supplementary material for: PFASUM: a substitution matrix from Pfam structural alignments
Source: BMC Bioinformatics. 2017 Jun 5;18:293. doi: 10.1186/s12859-017-1703-z (PMC5460430; doi:10.1186/s12859-017-1703-z)
Supplement: Supplementary file 5 — Table S1. Table of Standard Search Matrices with their relative entropy as listed in FASTA (Version: 36.3.8d, found in upam.h). Comparable PFASUM substitution matrices are listed with their respective entropy. Note, we list entries with n/a where no comparable PFASUM matrix can be found due to the distribution of entropies of possible PFASUM matrices. No matrix entropy is listed for OPTIMA5 and MD matrices. (PDF 42.6 kb) [file 12859_2017_1703_MOESM5_ESM.pdf]

Additional table 1: Table of *Standard Search Matrices* with their relative entropy as listed in `FASTA` (Version: 36.3.8d, found in `upam.h`). Comparable PFASUM substitution matrices are listed with their respective entropy. Note, we list entries with n/a where no comparable PFASUM matrix can be found due to the distribution of entropies of possible PFASUM matrices. No matrix entropy is listed for OPTIMA5 and MD matrices.

matrix	rel. entropy (bit)	matrix	rel. entropy (bit)
BLOSUM50	0.4808	PFASUM59	0.4849
BLOSUM62	0.6979	PFASUM78	0.6931
BLOSUM80	0.9868	n/a	n/a
PAM120	0.9790	n/a	n/a
PAM250	0.3540	PFASUM45	0.3529
VTML10	3.4680	n/a	n/a
VTML20	2.9125	n/a	n/a
VTML40	2.2675	n/a	n/a
VTML80	1.4279	n/a	n/a
VTML160	0.5625	PFASUM67	0.5649
VTML200	0.4121	PFASUM51	0.4084
