# Supplementary material for: PFASUM: a substitution matrix from Pfam structural alignments
Source: BMC Bioinformatics. 2017 Jun 5;18:293. doi: 10.1186/s12859-017-1703-z (PMC5460430; doi:10.1186/s12859-017-1703-z)
Supplement: Supplementary file 6 — Table S2. Table of gap parameters used for matrices at similar entropy levels that resulted in the highest coverage. (PDF 32.2 kb) [file 12859_2017_1703_MOESM6_ESM.pdf]

Additional table 2: Table of gap parameters used for matrices at similar entropy levels that resulted in the highest coverage.

Matrix	Database	gap open penalty	gap extension penalty	Coverage
BLOSUM50	ASTRAL20	-15	-1	0.1474
PFASUM59	ASTRAL20	-16	-1	0.1688
BLOSUM62	ASTRAL20	-10	-1	0.1465
PFASUM78	ASTRAL20	-9	-1	0.1546
PAM250	ASTRAL20	-9	-3	0.1105
PFASUM45	ASTRAL20	-14	-1	0.1658
VTML160	ASTRAL20	-16	-1	0.1566
PFASUM67	ASTRAL20	-15	-1	0.1587
VTML200	ASTRAL20	-14	-1	0.1598
PFASUM51	ASTRAL20	-15	-1	0.1672
BLOSUM50	ASTRAL40	-11	-2	0.4371
PFASUM59	ASTRAL40	-11	-2	0.4416
BLOSUM62	ASTRAL40	-10	-1	0.4346
PFASUM78	ASTRAL40	-10	-1	0.4372
PAM250	ASTRAL40	-10	-2	0.4024
PFASUM45	ASTRAL40	-13	-1	0.4448
VTML160	ASTRAL40	-15	-1	0.4386
PFASUM67	ASTRAL40	-9	-3	0.4388
VTML200	ASTRAL40	-14	-1	0.4392
PFASUM51	ASTRAL40	-17	-1	0.4418
BLOSUM50	ASTRAL70	-15	-1	0.5420
PFASUM59	ASTRAL70	-14	-1	0.5449
BLOSUM62	ASTRAL70	-9	-1	0.5402
PFASUM78	ASTRAL70	-9	-1	0.5433
PAM250	ASTRAL70	-11	-2	0.5187
PFASUM45	ASTRAL70	-10	-2	0.5484
VTML160	ASTRAL70	-11	-2	0.5448
PFASUM67	ASTRAL70	-10	-2	0.5433
VTML200	ASTRAL70	-9	-2	0.5459
PFASUM51	ASTRAL70	-10	-2	0.5460
