# Supplementary material for: PFASUM: a substitution matrix from Pfam structural alignments
Source: BMC Bioinformatics. 2017 Jun 5;18:293. doi: 10.1186/s12859-017-1703-z (PMC5460430; doi:10.1186/s12859-017-1703-z)
Supplement: Supplementary file 7 — Table S3. Table of Z-score values for the performance comparison of Standard Search Matrices with their PFASUM counterparts based on relative entropy. Non-significant Z-scores based on the 95% percentile are highlighted as bold. (PDF 43.0 kb) [file 12859_2017_1703_MOESM7_ESM.pdf]

Additional table 3: Table of Z-score values for the performance comparison of *Standard Search Matrices* with their PFASUM counterparts based on relative entropy. Non-significant Z-scores based on the 95% percentile are highlighted as bold.

SSEARCH Matrix	PFASUM	ASTRAL 20	ASTRAL 40	ASTRAL 70
BLOSUM50	PFASUM59	122.3182	23.8432	15.5655
BLOSUM62	PFASUM78	49.6553	12.2135	18.6066
PAM250	PFASUM45	351.3204	222.2383	169.4170
VTML160	PFASUM67	12.0249	**0.04165**	-8.4868
VTML200	PFASUM51	45.8990	12.2529	**-1.1018**
