# Supplementary material for: PFASUM: a substitution matrix from Pfam structural alignments
Source: BMC Bioinformatics. 2017 Jun 5;18:293. doi: 10.1186/s12859-017-1703-z (PMC5460430; doi:10.1186/s12859-017-1703-z)
Supplement: Supplementary file 8 — Table S4. Table of all substitution matrices in the homology search evaluation showing their highest coverage and their corresponding gap parameters for all three databases. (PDF 43.0 kb) [file 12859_2017_1703_MOESM8_ESM.pdf]

Additional table 4: Table of all substitution matrices in the homology search evaluation showing their highest coverage and their corresponding gap parameters for all three databases.

Matrix	Database	gap open penalty	gap ext. penalty	Coverage	Database	gap open penalty	gap ext. penalty	Coverage	Database	gap open penalty	gap ext. penalty	Coverage
PFASUM31	ASTRAL20	15	-2	0.1562	ASTRAL40	17	-1	0.4427	ASTRAL70	13	-2	0.5508
PFASUM43	ASTRAL20	14	-1	0.1650	ASTRAL40	13	-1	0.4448	ASTRAL70	12	-1	0.5479
PFASUM60	ASTRAL20	16	-1	0.1706	ASTRAL40	15	-1	0.4412	ASTRAL70	11	-2	0.5448
BLOSUM50	ASTRAL20	15	-1	0.1474	ASTRAL40	11	-2	0.4371	ASTRAL70	15	-1	0.5420
BLOSUM62	ASTRAL20	10	-1	0.1465	ASTRAL40	10	-1	0.4346	ASTRAL70	9	-1	0.5402
BLOSUM80	ASTRAL20	8	-1	0.1231	ASTRAL40	9	-1	0.4140	ASTRAL70	8	-1	0.5256
MD10	ASTRAL20	6	-2	0.0206	ASTRAL40	7	-2	0.1923	ASTRAL70	6	-2	0.3494
MD20	ASTRAL20	6	-2	0.0320	ASTRAL40	7	-2	0.2390	ASTRAL70	7	-2	0.3889
MD40	ASTRAL20	8	-2	0.0512	ASTRAL40	9	-2	0.2996	ASTRAL70	9	-2	0.4381
OPTIMA5	ASTRAL20	19	-3	0.1563	ASTRAL40	14	-3	0.4383	ASTRAL70	17	-3	0.5442
PAM120	ASTRAL20	9	-1	0.0988	ASTRAL40	9	-1	0.3976	ASTRAL70	10	-1	0.5092
PAM250	ASTRAL20	9	-3	0.1105	ASTRAL40	10	-2	0.4024	ASTRAL70	11	-2	0.5187
VTML10	ASTRAL20	5	-1	0.0192	ASTRAL40	6	-1	0.1909	ASTRAL70	5	-1	0.3476
VTML20	ASTRAL20	5	-1	0.0331	ASTRAL40	6	-1	0.2468	ASTRAL70	6	-1	0.3937
VTML40	ASTRAL20	7	-1	0.0557	ASTRAL40	7	-1	0.3162	ASTRAL70	7	-1	0.4472
VTML80	ASTRAL20	8	-1	0.0951	ASTRAL40	9	-1	0.3890	ASTRAL70	8	-1	0.5044
VTML120	ASTRAL20	9	-1	0.1310	ASTRAL40	9	-1	0.4260	ASTRAL70	9	-1	0.5337
VTML160	ASTRAL20	16	-1	0.1566	ASTRAL40	15	-1	0.4386	ASTRAL70	11	-2	0.5448
VTML200	ASTRAL20	14	-1	0.1598	ASTRAL40	14	-1	0.4392	ASTRAL70	9	-2	0.5459
