# Supplementary material for: PFASUM: a substitution matrix from Pfam structural alignments
Source: BMC Bioinformatics. 2017 Jun 5;18:293. doi: 10.1186/s12859-017-1703-z (PMC5460430; doi:10.1186/s12859-017-1703-z)
Supplement: Supplementary file 9 — Table S5. Table of Z-score values for the comparison between PFASUM Search Matrices and Standard Search Matrices on the three different ASTRAL datasets. Z-scores with |Z|≥1.96 represent statistically significant underlying distributions at the 95% confidence interval. Non-significant Z-scores are highlight in red. (PDF 60.6 kb) [file 12859_2017_1703_MOESM9_ESM.pdf]

Additional table 5: Table of Z-score values for the comparison between *PFASUM Search Matrices* and *Standard Search Matrices* on the three different ASTRAL datasets. Z-scores with $|Z| \geq 1.96$ represent statistically significant underlying distributions at the 95% confidence interval. Non-significant Z-scores are highlight in red.

		BLOSUM50	BLOSUM62	BLOSUM80	MD10	MD20	MD40	OPTIMA5	PAM120	PAM250	VTML10	VTML20	VTML40	VTML80	VTML120	VTML160	VTML200	PFASUM31	PFASUM43	PFASUM60
ASTRAL70	PFASUM31	49.87	62.02	143.79	1125.31	905.95	648.85	37.38	229.92	182.64	1183.97	854.69	572.76	272.88	98.63	34.94	26.75	0.00	17.42	34.96
	PFASUM43	33.53	45.96	130.36	1138.22	913.05	649.60	20.79	219.02	170.28	1200.51	859.63	570.82	262.94	83.79	18.69	10.18	-17.42	0.00	18.10
	PFASUM60	15.50	27.78	112.45	1118.18	893.55	630.14	2.91	201.42	152.24	1179.30	840.87	552.36	244.00	65.75	**1.29**	-7.33	-34.96	-18.10	0.00
ASTRAL40	PFASUM31	29.28	39.56	145.18	1378.02	1097.67	737.82	22.12	228.64	207.94	1363.20	1024.10	638.93	271.24	83.25	20.86	17.50	0.00	-10.51	9.44
	PFASUM43	40.45	50.85	158.76	1425.60	1136.37	765.42	33.09	244.26	223.43	1408.99	1059.21	663.47	287.76	95.73	32.21	28.72	10.51	0.00	20.64
	PFASUM60	21.09	31.86	142.02	1440.62	1142.94	762.01	13.64	229.21	207.73	1422.68	1062.97	657.55	273.66	77.38	12.05	8.58	-9.44	-20.64	0.00
ASTRAL20	PFASUM31	51.26	57.54	203.63	1061.17	955.94	757.71	**-1.70**	363.50	294.64	1070.21	944.53	706.49	386.25	148.43	-2.74	-20.92	0.00	-51.14	-80.63
	PFASUM43	101.83	108.95	256.11	1116.56	1011.97	813.08	50.27	416.42	348.98	1125.43	1000.55	761.17	438.81	199.51	49.05	31.72	51.14	0.00	-30.83
	PFASUM60	129.70	137.01	280.25	1098.78	1000.60	814.22	80.21	435.32	370.41	1107.28	989.95	765.40	456.91	224.90	78.94	62.52	80.63	30.83	0.00
