# Supplementary material for: PFASUM: a substitution matrix from Pfam structural alignments
Source: BMC Bioinformatics. 2017 Jun 5;18:293. doi: 10.1186/s12859-017-1703-z (PMC5460430; doi:10.1186/s12859-017-1703-z)
Supplement: Supplementary file 10 — Table S6. Average q-score $\bar{q}$q¯ for the SABmark alignments, split between superfamily alignments and so-called “twilight zone” alignments. Matrices with the highest performances are highlighted in bold. (PDF 47.5 kb) [file 12859_2017_1703_MOESM10_ESM.pdf]

Additional table 6: Average q-score $\bar{q}$ for the SABmark alignments, split between superfamily alignments and so-called "twilight zone" alignments. Matrices with the highest performances are highlighted in bold.

	Matrix	average $\mathcal{Q}$-score
Twilight Zone	BLOSUM50	0.3913
	BLOSUM62	0.3914
	PAM250	0.3710
	PFASUM31	0.4034
	PFASUM43	0.4002
	**PFASUM60**	**0.4048**
	VTML160	0.3997
	VTML200	0.3885
Superfamilies	BLOSUM50	0.6342
	BLOSUM62	0.6343
	PAM250	0.6115
	**PFASUM31**	**0.6417**
	PFASUM43	0.6378
	PFASUM60	0.6407
	VTML160	0.6379
	VTML200	0.6381
